# DNA vaccines delivered by human papillomavirus pseudovirions as a promising approach for generating antigen-specific CD8+ T cell immunity

**DOI:** 10.1186/2045-3701-1-26

**Published:** 2011-07-28

**Authors:** Shiwen Peng, Barbara Ma, Shu-Hsia Chen, Chien-Fu Hung, TC Wu

**Affiliations:** 1 Department of Pathology, Johns Hopkins Medical Institutions, Baltimore, MD, USA; 2 Department of Obstetrics and Gynecology, Johns Hopkins Medical Institutions, Baltimore, MD, USA; 3 Department of Oncology, Johns Hopkins Medical Institutions, Baltimore, MD, USA; 4 Department of Molecular Microbiology and Immunology, Johns Hopkins Medical Institutions, Baltimore, MD, USA; 5 Department of Oncological Sciences, Mount Sinai School of Medicine, New York, NY, USA

## Abstract

**Background:**

Human papillomavirus (HPV) pseudovirions have recently been shown to deliver DNA efficiently *in vivo*, resulting in the priming of antigen-specific CD8+ T cells in vaccinated mice. In the current study, we compare the different preparation methods for the generation of HPV pseudovirions for their ability to efficiently infect cells. We also compare the antigen-specific CD8+ T cell immune responses generated by different DNA delivery methods and several commonly used forms of vaccination with that of HPV pseudovirions.

**Results:**

We found that the preparation method of pseudovirions is important for the efficient delivery of encapsidated DNA. We have shown that vaccination with DNA encoding model antigen ovalbumin (OVA) delivered by HPV-16 pseudovirions was capable of generating therapeutic antitumor effects against OVA-expressing tumor. In addition, vaccination with DNA encoding OVA delivered by HPV-16 pseudovirions generated the highest number of OVA-specific CD8+ T cells in mice in our system compared to DNA delivered by other delivery methods. We also found that vaccination with OVA DNA delivered by HPV-16 pseudovirions generated the highest number of OVA-specific CD8+ T cells in mice compared to other forms of antigen-specific vaccines. Furthermore, HPV-16 pseudovirions were capable of carrying DNA vaccine encoding clinically relevant antigen, telomerase reverse transcriptase, to generate antigen-specific CD8+ T cell immune responses.

**Conclusions:**

Our data suggest that DNA vaccines delivered by HPV-16 pseudovirions may be advantageous compared to other delivery methods and other forms of antigen-specific vaccines for application to antigen-specific immunotherapy.

## Background

DNA vaccination has emerged as a promising way to generate antigen-specific T cell immunity due to its safety, stability, and capacity for repeated administration. However, naked DNA vaccines suffer from limited vaccine potency due to poor transfection efficiency *in vivo*. Therefore, an optimized and efficient delivery system that improves the transfection efficiency of DNA vaccines into cells *in vivo *may significantly improve the antigen-specific immunity generated by DNA vaccination for the control of virus-associated infections and/or tumors.

We have recently introduced the use of replication-defective human papillomavirus (HPV) pseudovirions as a novel approach to improve naked DNA vaccine delivery *in vivo *[1]. DNA plasmids can be packaged into the papillomavirus L1 and L2 capsid proteins to generate a 'pseudovirion' that can efficiently deliver the encapsidated DNA into infected cells. The encapsulation of the therapeutic DNA vaccine protects the DNA from nucleases and provides efficient targeted delivery with great stability. Additionally, because HPV pseudovirions contain a DNA construct with genes of interest, but not the natural HPV viral genome, they are non-replicative and lack many of the safety concerns associated with live viral vectors. Furthermore, neutralizing antibodies against one type of papillomavirus pseudovirion are usually not cross-reactive to other types of papillomavirus pseudovirions. The spectrum of over 100 different types of papillomavirus pseudovirions allows for repeated boosting with different types of HPV pseudovirions without concern for preexisting immunity. Thus, HPV pseudovirions represent a potentially safe gene delivery method for clinical usage.

We previously characterized human papillomavirus pseudovirions as an efficient delivery system for DNA vaccines *in vivo *[1]. We demonstrated that vaccination with HPV-16 pseudovirions containing a DNA vaccine encoding model antigen, ovalbumin (OVA), (HPV-16/OVA psV) subcutaneously generated significantly stronger OVA-specific CD8+ T cell immune responses compared with OVA DNA vaccination via gene gun in a dose-dependent manner. We demonstrated that the L2 minor capsid protein was essential for the infectivity mediated by HPV-16/OVA psV. Additionally, we showed that papillomavirus pseudovirions are capable of infecting DCs [1]. Furthermore, the papillomavirus L1 capsid protein activates DCs to augment the immune response [2,3]. Thus, human papillomavirus pseudovirions represent an innovative and promising delivery system to trigger potent antigen-specific immune responses.

In the current study, we further characterize the application of HPV pseudovirions as an important method for the delivery of naked DNA immunization. We compared the method of preparing HPV pseudovirions for their ability to efficiently deliver DNA to cells. In addition, we analyzed the capability of HPV pseudovirions to deliver naked DNA to a bone marrow-derived dendritic cell line. Furthermore, we compared the delivery of DNA by HPV pseudovirions with other methods of administration and other forms of vaccines for their ability to generate antigen-specific CD8+ T cell immune responses. Our data indicate that the method of preparing HPV pseudovirion is crucial for their ability to infect cells. In addition, DNA vaccines delivered by HPV pseudovirions are able to effectively be delivered to dendritic cells, resulting in potent antigen-specific CD8+ T cell immune responses compared to different delivery methods and other forms of vaccination. The potential clinical applications of HPV pseudovirion technology for delivery of naked DNA vaccine are discussed.

## Results

### HPV pseudovirions prepared by intracellular assembly can infect cells with much greater efficiency than HPV pseudovirions prepared by *in vitro *assembly

The preparation method for pseudovirions may be crucial to the efficiency of DNA delivery. It has been previously shown that naked DNA can be encapsidated by L1 and L2 capsid proteins using *in vitro *assembly [4,5]. The HPV structural proteins can spontaneously self-assemble into virus-like particles (VLPs) that morphologically resemble the native virions. These pseudovirions generated by *in vitro *assembly involve the disruption and refolding of HPV L1L2 VLPs [4,5]. In general, the preparation of pseudovirions by *in vitro *assembly is technically demanding and the pseudovirion generated via this mechanism lacks L2, which is required for infection. In comparison, a recent method involving intracellular assembly of papillomaviral vectors was shown to generate high titers of replication-defective papillomavirus pseudovirions for vaccination [6,7]. This intracellular assembly method provides packaging of the target plasmid within an L1 and L2 capsid. The production cell line, 293 cells, is engineered to express high levels of SV40 large T antigen (293TT) to drive the amplification of the target plasmid containing an SV40 origin of replication. These 293TT cells are co-transfected with codon-optimized L1 and L2 capsid genes in an expression vector that is too large for its efficient encapsidation along with the target plasmid (which can contain the SV40 origin of replication but it is not required), allowing for efficient intracellular production of HPV pseudovirions encapsidating DNA.

**Figure 1:**
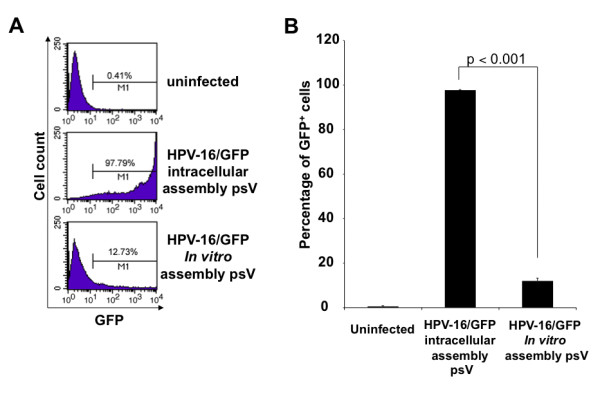
**Comparison of infectivity between HPV-16 pseudovirions carrying GFP prepared from intracellular assembly with HPV-16 pseudovirions carrying GFP generated from *in vitro *assembly**. HPV-16 pseudovirions carrying GFP (HPV-16/GFP psV) were prepared using two different methods (intracellular assembly versus *in vitro *assembly) as described in the **Materials and Methods**. For the characterization of infectivity, 1 × 10^5 ^of 293TT cells were seeded to each well of a 24-well plate the day before the infection. HPV-16/GFP psV (3 × 10^9 ^psV particles containing ~30 ng of pcDNA3-GFP plasmids) prepared from intracellular assembly or *in vitro *assembly were diluted with 1 ml of medium, added to each well and incubated at 37°C. After 72 hours, the cells were harvested, and GFP-positive cells were analyzed by flow cytometry analysis using a FACSCalibur flow cytometer and analyzed with CellQuest software. Uninfected cells served as a negative control. **A**. Representative flow cytometry data. **B**. Graphical representation of the percentage of GFP+ cells. Data expressed as means ± standard deviations (SD) are representative of at least two different experiments.

We therefore compared the infectivity of HPV-16 pseudovirions carrying GFP (HPV-16/GFP psV) prepared by *in vitro *assembly with the infectivity of HPV-16/GFP psV prepared by intracellular assembly. As shown in Figure 1, the infectivity of HPV-16/GFP psV prepared by *in vitro *assembly was significantly lower compared to that of HPV-16/GFP psV prepared by intracellular assembly. These data suggest that this new method for the generation of large quantities of replication-defective HPV pseudovirions is a significantly more efficient method in comparison to the preparation of HPV pseudovirions by *in vitro *assembly.

### HPV-16 pseudovirions can efficiently infect dendritic cell line *in vitro *in a dose-dependent manner

**Figure 2:**
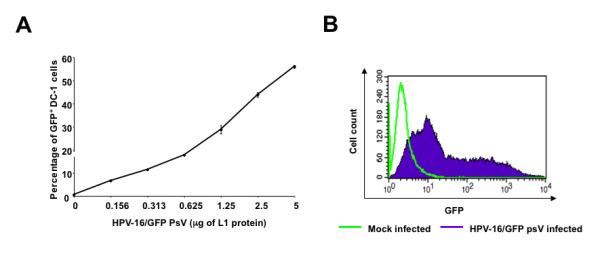
**Infection of dendritic cells by HPV-16 pseudovirions**. DC-1 cells (5 × 10^4 ^cells/well) were seeded into 24-well plate the night before infection. The seeded DC-1 cells were then infected with indicated amount of HPV16-GFP pseudoviruses (total amount of L1 protein) for 72 hours and GFP-positive cells were examined by flow cytometry. **A**. Line graph of percentage of GFP+ cells. **B**. Representative flow cytometry data of DC-1 cells infected with HPV-16/GFP psV (5 ug of L1 protein).

We have previously demonstrated that FITC-labeled HPV-16 pseudovirions carrying DNA encoding model antigen ovalbumin (HPV-16/OVA psV) were able to be taken up by CD11c+ dendritic cells in the draining lymph nodes of vaccinated mice [1]. To determine whether the dose of HPV pseudovirions correlates with the infectivity of DC-1 dendritic cell line, 5 × 10^4 ^of DC-1 cells/well were seeded into 24-well plates the night before infection. The seeded DC-1 cells were then infected with increasing amounts of HPV-16/GFP psV (L1 protein ranging from 0 ug to 5 ug) for 72 hours and GFP expression was examined by flow cytometry. There was a clear correlation between percentage of GFP+ DC-1 cells and amount of L1 protein in HPV-16/GFP psV, as demonstrated by the increase in GFP expression in DC-1 cells with increasing amount of L1 protein in HPV-16/GFP psV (Figure 2A). As shown in Figure 2B, DC-1 cells infected with HPV-16/GFP psV (5 ug L1 protein) showed a clear shift in the peak of GFP expression compared to that of uninfected cells, indicating that the majority of cells infected with HPV-16/GFP psVs had significantly greater GFP expression than uninfected cells. Thus, our data suggest that HPV pseudovirions can efficiently infect dendritic cells *in vitro *in a dose-dependent manner.

### Treatment of tumor-bearing mice with DNA delivered by HPV-16 pseudovirions generates therapeutic antitumor effects

**Figure 3:**
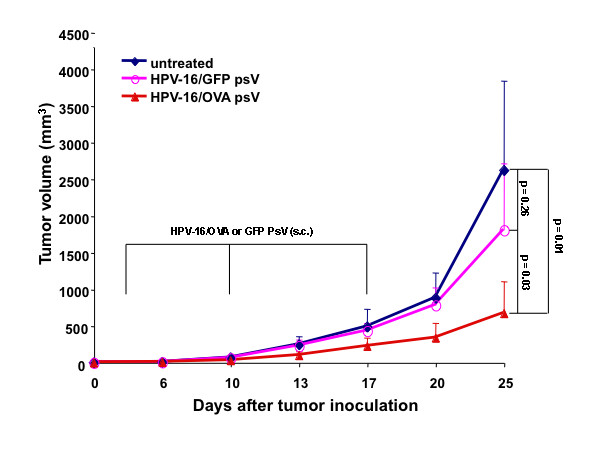
**In vivo tumor treatment experiment with HPV-16 pseudovirion vaccination**. 5-8 weeks old C57BL/6 mice (5 mice per group) were injected with 1 × 10^5 ^B16/OVA tumor cells subcutaneously. 3 days after tumor cell injection, the mice were vaccinated with either 5 μg (total L1 protein amount) of HPV-16/OVA, or HPV-16/GFP pseudovirions via footpad injection. The mice were boosted with the same regimen on day 10 and 17. Tumor growth was monitored twice a week and tumor volume was calculated as described in the Material and Methods section.

We have previously demonstrated that C57BL/6 mice vaccinated with HPV-16 pseudovirions carrying OVA DNA were capable of preventing tumor growth upon challenge with OVA-expressing tumor [1]. To determine if DNA delivered by HPV-16 pseudovirions could generate appreciable therapeutic antitumor effects, we performed *in vivo *tumor treatment experiments. C57BL/6 mice were inoculated subcutaneously with B16/OVA tumor cells and treated with the various vaccination groups three days later. Mice were boosted with the same regimen on day 10 and 17 after tumor inoculation. As shown in Figure 3, tumor-bearing mice treated with HPV-16/OVA psV demonstrated significantly reduced tumor volume as compared to tumor-bearing mice treated with HPV-16 pseudovirions carrying DNA encoding irrelevant protein (GFP) or untreated mice. Thus, our data suggest that treatment with HPV-16 pseudovirions carrying OVA DNA can generate therapeutic antitumor effects against OVA-expressing tumors in tumor-bearing mice.

### Vaccination with DNA delivered by HPV-16 pseudovirions generates the highest levels of antigen-specific CD8+ T cell immune responses compared to vaccination with DNA delivered by other methods

**Figure 4:**
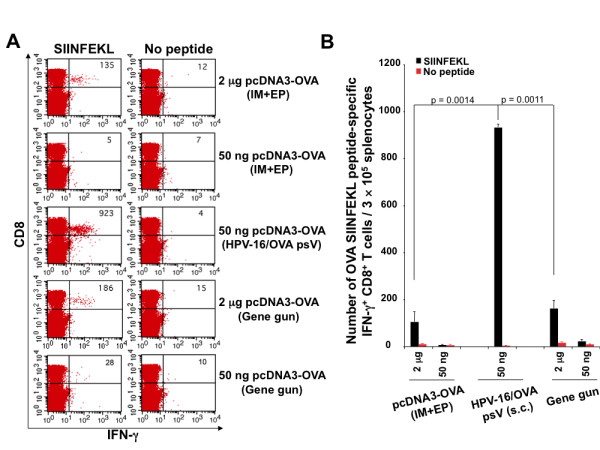
**Comparison of OVA-specific CD8+ T cell responses induced by pcDNA3-OVA delivered by different methods of administration**. 5 to 8 weeks old C57BL/6 mice (5 per group) were vaccinated with HPV-16/OVA pseudovirions containing pcDNA3-OVA (50 ng) subcutaneously (s.c.), or pcDNA3-OVA either intramuscularly (IM) by electroporation (EP) (2 ug or 50 ng) or intradermally by gene gun delivery (2 ug or 50 ng). These vaccinated mice were boosted once after 7 days with the same dose and regimen. Splenocytes were collected one week after last vaccination, and stimulated with OVA MHC class I peptide SIINFEKL (1 ug/ml) in the presence of GolgiPlug. The OVA-specific CD8^+ ^T cells were then analyzed by staining surface CD8 and intracellular IFN-γ. **A**. Representative flow cytometry data. **B**. Graphical representation of the number of OVA-specific CD8+ T cells per 3 × 10^5 ^splenocytes. Data expressed as means ± standard deviations (SD) are representative of at least two different experiments.

We next compared the OVA-specific CD8+ T cell immune responses generated by vaccination with OVA-specific DNA vaccines delivered by different methods including intramuscular injection followed by electroporation, gene gun, and HPV-16 pseudovirions. As shown in Figure 4, C57BL/6 mice vaccinated subcutaneously with HPV-16/OVA psV generated the highest number of OVA MHC class I peptide (SIINFEKL)-specific CD8+ T cell immune responses among all vaccination groups. In addition, we observed that DNA vaccine delivered by intramuscular injection followed by electroporation and DNA vaccine delivered by gene gun both generated higher OVA-specific CD8+ T cell immune responses at a higher dose of DNA (2 ug) compared to a lower dose of DNA (50 ng). Furthermore, we observed that delivery of DNA vaccine by HPV-16 pseudovirions generated a significantly higher antigen-specific CD8+ T cell immune response even with a lower dose of DNA vaccine contained in the pseudovirion. These data suggest that antigen-specific vaccination with DNA vaccine delivered by pseudovirion represents one of the most promising approaches for generating antigen-specific CD8+ T cell immune responses compared to other methods of delivering DNA *in vivo*.

### HPV-16 pseudovirions generate the highest level of antigen-specific CD8+ T cell immune responses compared to other forms of antigen-specific vaccines

**Figure 5:**
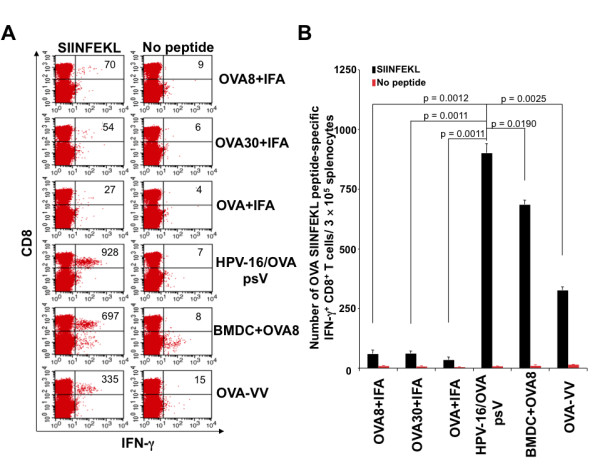
**Comparison of OVA-specific CD8+ T cell responses induced by different forms of OVA-specific vaccines**. 5 to 8 weeks old C57BL/6 mice (5 per group) were vaccinated with HPV-16 pseudovirions carrying OVA (HPV-16/OVA psV) containing pcDNA3-OVA (50 ng) subcutaneously (s.c.), or OVA8 (aa257-264) (15 μg) in incomplete Freund's adjuvant (IFA), or OVA30 (aa241-270) (50 μg) in IFA, or OVA protein (50 μg) in IFA s.c., or OVA8-pulsed bone marrow-derived dendritic cells (BMDC) (5 × 10^5 ^cells/mouse). Mice were boosted once after 7 days with the same dose and regimen. At the time of boost, another group of naïve mice were vaccinated with OVA-expressing vaccinia viruses (OVA-VV) (1 × 10^7 ^pfu) intraperitoneally. Splenocytes were collected one week after last vaccination, and stimulated with OVA MHC class I peptide SIINFEKL (1 μg/ml) at the presence of GolgiPlug. The OVA-specific CD8^+ ^T cells were then analyzed by staining surface CD8 and intracellular IFN-γ. **A**. Representative flow cytometry data. **B**. Graphical representation of the number of OVA-specific CD8+ T cells per 3 × 10^5 ^splenocytes. Data expressed as means ± standard deviations (SD) are representative of at least two different experiments.

In addition to the many vaccine delivery methods that are currently being explored, there exist many forms of vaccination for antigen-specific immunotherapy. It is important to evaluate the efficacy of DNA vaccines delivered by HPV pseudovirions compared to other forms of antigen-specific vaccines for their ability to generate antigen-specific CD8+ T cells. We therefore compared the antigen-specific CD8+ T cell immune responses generated by HPV-16/OVA psV with other forms of vaccination including peptide-based (OVA8 (aa257-264) in incomplete Freund's adjuvant (IFA) and OVA30 (aa241-270) in IFA), protein-based (OVA protein in IFA), dendritic cell-based (OVA8-pulsed BMDCs) and vaccinia viral vector-based vaccination expressing OVA (OVA-VV). As shown in Figure 5, mice vaccinated subcutaneously with HPV-16/OVA psV generated the highest level of OVA-specific CD8+ T cell immune responses among all forms of OVA-specific vaccines. These data support that antigen-specific vaccination with DNA vaccine delivered by pseudovirion generates the best antigen-specific CD8+ T cell immune responses compared to other forms of antigen-specific vaccines.

### HPV pseudovirions is capable of delivering DNA encoding clinically relevant antigen to generate enhanced antigen-specific CD8+ T cell immune responses

**Figure 6:**
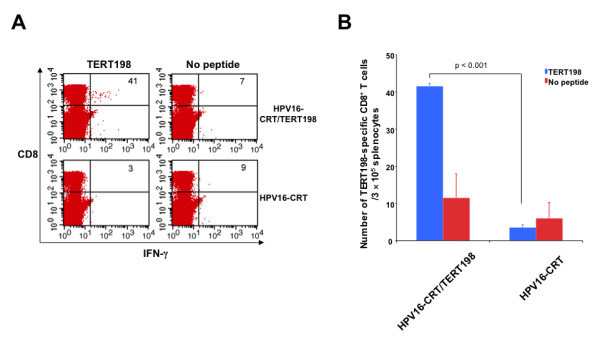
**Intracellular cytokine staining followed by flow cytometry analysis for TERT198-specific CD8+ T cell immune responses**. 5-8 weeks old C57BL/6 mice were vaccinated with 5 μg of L1 protein of HPV16-CRT/TERT198, or HPV16-CRT pseudovirions via footpad injection, and boosted twice at 4-day interval with the same regimen. 1 week after last vaccination, splenocytes were prepared and stimulated with TERT198 peptide (1 μg/ml) at the presence of GolgiPlug overnight at 37 C. The TERT198-specific CD8+ T cells were then analyzed by staining surface CD8 and intracellular IFN-γ. **A**. Representative flow cytometry data. **B**. Graphical representation of the number of TERT198-specific CD8+ T cells per 3 × 10^5 ^splenocytes. Data expressed as means ± standard deviations (SD) are representative of at least two different experiments.

In order to explore whether DNA delivered by HPV pseudovirions can be applied to clinically relevant antigens, we have chosen telomerase reverse transcriptase (TERT) (for review, see [8]). TERT is an endogenous antigen that is usually not expressed in most normal human somatic tissues but is reactivated in 85% of tumors. Upon reactivation of telomerase in tumor cells, TERT is processed and presented on the MHC class I molecules of tumor cells. Hence, TERT represents an attractive target for vaccine development. We therefore created DNA encoding calreticulin (CRT) linked to TERT198 minigene (aa198-205, VGRNFTNL) [9] delivered by HPV pseudovirions. CRT has been shown to be one of the most potent intracellular targeting strategies to enhance antigen-specific CD8+ T cell immune responses in our previous study [10]. TERT198 minigene was shown to have a significantly high avidity for binding with H-2K^b ^[9]. As shown in Figure 6, mice vaccinated with HPV-16 pseudovirions carrying CRT linked to TERT198 minigene (HPV-16-CRT/TERT198) demonstrated significantly higher number of TERT198-specific CD8+ T cell immune responses compared to mice vaccinated with control HPV-16 pseudovirions carrying CRT DNA. Thus, the delivery system of HPV-16 pseudovirions can be applied to other DNA vaccines targeting clinically relevant antigenic systems.

## Discussion

In the current study, we found that replication-defective HPV pseudovirions prepared by intracellular assembly can infect cells with greater efficiency than HPV pseudovirions prepared by *in vitro *assembly. Our data also indicate that HPV-16 pseudovirions are capable of infecting DC-1 dendritic cell line *in vitro*. We also demonstrate that tumor-bearing mice treated with HPV-16/OVA psV could generate therapeutic antitumor effects against OVA-expressing tumors. In addition, we demonstrate that HPV pseudovirions carrying DNA encoding antigen generate the highest levels of antigen-specific CD8+ T cell immune responses compared to DNA delivered by other methods, including gene gun and intramuscular injection followed by electroporation. We also show that HPV pseudovirions generate the highest level of antigen-specific CD8+ T cell immune responses compared to other forms of antigen-specific vaccines. Furthermore, HPV pseudovirions could be used to deliver clinically relevant antigen for the generation of antigen-specific CD8+ T cell immune responses.

We observed that there is significantly higher infectivity in HPV pseudovirions generated by intracellular assembly compared to HPV pseudovirions prepared by *in vitro *assembly (Figure 1). The method of HPV pseudovirion generated by intracellular assembly resembles a system more biologically similar to the natural environment for viral assembly. Therefore, HPV pseudovirions generated by intracellular assembly will likely be more efficient at packaging the naked DNA into the L1 and L2 capsid proteins versus that of HPV pseudovirions generated by *in vitro *assembly. Furthermore, the conformation of L1 and L2 capsid proteins in the HPV pseudovirions generated by intracellular assembly will likely be better preserved compared to HPV pseudovirions generated by *in vitro *assembly. The *in vitro *assembly requires the disruption of L1 and L2 capsid proteins and refolding, a process during which may result in a conformational change of L1 and L2 capsid proteins. It has been shown that L2 is essential for the infectivity of the HPV pseudovirion [1,11,12]. These factors may account for the poorer infectivity of HPV pseudovirions generated by *in vitro *assembly compared to HPV pseudovirions generated by intracellular assembly.

Intradermal administration by gene gun and intramuscular injection followed by electroporation represent two state-of-the-art methods for DNA vaccine delivery. There have also been several clinical trials using gene gun or electroporation for therapeutic HPV DNA vaccines [13-15]. It is therefore important for future clinical translation to perform a head-to-head comparison to determine whether vaccination with HPV pseudovirion carrying DNA will be better than the aforementioned DNA vaccine delivery techniques at generating robust antigen-specific CD8+ T cell immune responses. We have previously compared the antigen-specific immune responses generated by HPV-16-pseudovirion carrying DNA encoding model antigen OVA with naked DNA vaccine encoding the same model antigen administered by gene gun [1]. Mice vaccinated with HPV-16/OVA pseudovirions generated significantly higher number of OVA-specific CD8+ T cell immune responses compared to mice vaccinated with naked OVA DNA via gene gun. In the current study, we found that vaccination with HPV-16/OVA pseudovirions generated the highest levels of antigen-specific CD8+ T cell immune responses compared to intradermal administration via gene gun and intramuscular injection followed by electroporation (Figure 4). Furthermore, delivery of DNA vaccine by HPV-16/OVA pseudovirions generated a significantly greater OVA-specific CD8+ T cell immune response even with a lower dose of OVA DNA contained in the pseudovirion. Thus, DNA vaccine delivered by pseudovirion represents a more potent method to deliver naked DNA vaccine *in vivo *compared to gene gun and electroporation for their ability to generate antigen-specific immune responses.

We also compared DNA vaccine delivered by pseudovirions to other forms of antigen-specific vaccines including peptide-based vaccination, protein-based vaccination, dendritic cell-based vaccination and viral vector-based vaccination. We found that HPV-16/OVA pseudovirions generated the highest level of OVA-specific CD8+ T cell immune responses compared to other established forms of vaccination in the conditions tested (Figure 5). However, one of the major limitations for such kind of approach is that the forms of vaccines compared may not be optimized, including the conditions used for HPV pseudovirions. Therefore, the optimization of vaccine potency for each form of the vaccine before we perform a head-to-head comparison in the future will generate a more comprehensive picture of the efficacy of the different vaccination approaches compared to HPV pseudovirions.

There are several points of consideration for the clinical translation of HPV pseudovirions (for review, see [16]). For example, it is important to consider the type of HPV pseudovirion used to deliver the DNA vaccine. Currently, the commercially available prophylactic HPV vaccines use virus-like particles (VLPs) that include HPV types 16, 18, and/or HPV types 6 and 11. Vaccinations with HPV VLPs have been shown to generate potent type-specific neutralizing antibodies, which can inhibit subsequent infection of the same type of human papillomavirus. Thus, to avoid inhibition of vaccine efficacy by pre-existing immunity with this preventive HPV vaccine, it is essential to consider a different type of papillomavirus for pseudovirion delivery of DNA vaccine. This has broad clinical implications for delivering therapeutic HPV DNA vaccines.

## Conclusions

In summary, HPV pseudovirions carrying DNA vaccine represent a significantly more efficient system compared to other methods of DNA vaccine delivery and other forms of vaccination for generating antigen-specific immunity. DNA vaccines delivered by HPV pseudovirions combine both the safety features of naked DNA and the potent infectivity of viral vector vaccines without the disadvantages associated with each of them. Thus, gene delivery using HPV pseudovirion technology represents a potentially promising non-viral gene delivery system to trigger potent immune responses against viral infections and cancer.

## Methods

### Mice

C57BL/6 mice (5-to-8-weeks old) were purchased from National Cancer Institute (Frederick, MD, USA). All animals were maintained under specific pathogen-free conditions, and all procedures were performed according to approved protocols and in accordance with recommendations for the proper use and care of laboratory animals.

### Cells

293TT cells were generated by transfecting 293T cells with an additional copy of the SV40 large T antigen and were kindly provided by J. Schiller (NCI, NIH) [6]. 293TT cells were grown in complete Dulbecco's modified Eagle medium (DMEM) (Invitrogen) containing 10% heat-inactivated fetal bovine serum (Gemini Bio-Products). DC-1 dendritic cell line has been described previously [17].

### Peptides, antibodies and reagents

The H-2K^b^-restricted Ovalbumin (OVA) peptide, SIINFEKL, was synthesized by Macromolecular Resources (Denver, CO) at a purity of ≥ 80%. FITC-conjugated rat anti-mouse IFN-γ, PE-conjugated anti-mouse CD8, PE-Cy5 conjugated anti-mouse B220, and APC-conjugated anti-mouse CD11c antibodies were purchased from BD Pharmingen (BD Pharmingen, San Diego, CA). A horseradish peroxidase-conjugated rabbit anti-mouse immunoglobulin G (IgG) antibody was purchased from Zymed (San Francisco, CA). Peptides were generated as described before [18]. The following dominant minimal CTL peptide was used: OVA aa257-264 (OVA8). In addition, the long peptide deduced from the natural sequence of OVA protein was used: CTL peptide OVA aa241-270 SMLVLLPDEVSGLEQLESIINFEKLTEWTS (OVA30). OVA protein was purchased from Sigma. Incomplete Freund's adjuvant was purchased from Difco Laboratories. The vaccinia virus expressing the full-length chicken ovalbumin (OVA) was generated using methods described previously [19,20].

### Plasmid construction

The plasmids encoding HPV16 and L1 and L2 (pShell16, p16L1 and p16L2) were kindly provided by Dr. John Schiller (NCI). The generation of ovalbumin-expressing plasmid (pcDNA3-OVA) and GFP-expressing plasmid (pcDNA3-GFP) has been described previously [21,22]. The generation of pcDNA3-CRT has been described previously [23]. For the generation of pcDNA3-CRT/TERT198, the synthesized oligos (AATTCgtgggcaggaatttcactaacctttgaA and AGCTTtcaaaggttagtgaaattcctgcccacG) were annealed and subsequently cloned into EcoRI and HindIII sites of pcDNA3-CRT. The accuracy of the constructs were confirmed by DNA sequencing.

### HPV pseudovirion production

For the generation of HPV pseudovirions using *in vitro *assembly, 293TT cells were transfected with pShell plasmid expressing codon-optimized HPV-16 L1, L2 capsid proteins only (without pcDNA3-GFP) using previously described protocols. The HPV structural capsid proteins have the ability to self-assemble into virus-like particles (VLPs). *In vitro *assembly into HPV pseudovirions involves the disruption and refolding of HPV-16 L1L2 VLPs [4,5]. Briefly, 5 μg of purified HPV-16 L1L2 VLPs were incubated in 50 mM Tris-HCL buffer (pH 7.5) containing 150 mM NaCl, 10 mM EGTA and 20 mM dithiothreitol (DTT) in a final volume of 100 μl at room temperature (RT) for 30 minutes. 1 μg of pcDNA3-GFP plasmid in 50 mM Tris-HCL buffer and 150 mM NaCl was added to the disrupted VLPs at this step. The preparations were diluted with 25 mM CaCl_2 _and 20% dimethyl sulfoxide in equal volume at RT for 1 hour, and then treated with 10 U of Benzonase Nuclease for 1 hour at RT to remove un-encapsidated plasmids.

For the generation of HPV pseudovirions by intracellular assembly, HPV-16 pseudovirions were made as described previously [6]. Briefly, 293TT cells were co-transfected with pShell plasmid expressing codon-optimized HPV-16 L1, L2 proteins and pcDNA3-GFP using Lipofectamine 2000 (Invitrogen, Carlsbad, CA). After 44 hours incubation, the cells were harvested and washed with Dulbecco's PBS (Invitrogen) supplemented with 9.5 mM MgCl_2 _and antibiotic-antimycotic mixture (DPBS-Mg) (Invitrogen). The cells were suspended in DPBS-Mg supplemented with 0.5% Briji58, 0.2% Benzonase (Novagen), 0.2% Plasmid Safe (Epicentre) at > 100 × 10^6 ^cells/ml and incubated at 37°C for 24 hours for capsid maturation. After maturation, the cell lysate was chilled on ice for 10 minutes. The salt concentration of the cell lysate was adjusted to 850 mM and incubated on ice for 10 minutes. The lysate was then clarified by centrifugation, and the supernatant was then layered onto an Optiprep gradient. The gradient was spun for 4.5 hours at 16°C at 40,000 rpm in a SW40 rotor (Beckman). The purity of HPV pseudovirions was evaluated by running the fractions on 4-15% gradient SDS-PAGE gel. The encapsulated DNA plasmid was quantified by extracting encapsidated DNA from Optiprep factions followed by quantitative real time PCR compared to serial dilutions of naked DNA as described in [1]. The concentration of pcDNA3 plasmid DNA and pcDNA3-OVA DNA in the pseudovirions was determined to be approx. 6.2 ng of DNA per 1 μg of L1 protein.

### Generation of bone marrow-derived dendritic cells

Bone marrow-derived dendritic cells (BMDCs) were generated from bone marrow progenitor cells as described previously [24]. Briefly, bone marrow cells were flushed from the femurs and tibiae of 5- to 8-week-old C57BL/6 mice. Cells were washed twice with RPMI-1640 after lysis of red blood cells and resuspended at a density of 1 × 10^6^/ml in RPMI-1640 medium supplemented with 2 m*M *glutamine, 1 m*M *sodium pyruvate, 100 m*M *nonessential amino acids, 55 μ*M *β-mercaptoethanol, 100 IU/ml penicillin, 100 g/ml streptomycin, 5% fetal bovine serum, and 20 ng/ml recombinant murine GM-CSF (PeproTech, Rock Hill, NJ). The cells were then cultured in a 24-well plate (1 ml/well) at 37°C in 5% humidified CO_2_. The wells were replenished with fresh medium supplemented with 20 ng/ml recombinant murine GM-CSF on days 2 and 4. The cells were harvested as indicated.

### In vitro infection with HPV pseudovirions

DC-1 cells (5 × 10^4 ^cells/well) were seeded into 24-well plate the night before infection. The seeded DC-1 cells were then infected with HPV16-GFP pseudovirions (L1 protein amount ranging from 0 ug to 5 ug). 72 hours later, the cells were analyzed for GFP expression by flow cytometry.

### Vaccination with HPV pseudovirions

C57BL/6 mice (5 per group) were vaccinated with indicated HPV pseudovirions (adjusted to 5 μg L1 protein amount) subcutaneous injection at both hind footpads. 7 days later, the mice were boosted with indicated HPV pseudovirions with the same dose and regimen. For antigen-specific T cell detection, mouse splenocytes were harvested 1 week after last vaccination.

### Intradermal DNA vaccination via gene gun

Gene gun particle-mediated DNA vaccination was performed as described previously [25]. Gold particles coated with pcDNA3-OVA or pcDNA3 were delivered to the shaved abdominal regions of mice by using a helium-driven gene gun (Bio-Rad Laboratories Inc., Hercules, Calif.) with a discharge pressure of 400 lb/in^2^. C57BL/6 mice (5 per group) were immunized with 2 μg of the DNA vaccine and boosted with the same dose and regimen 1 week later. Splenocytes were harvested 1 week after the last vaccination.

### Intramuscular DNA vaccination with electroporation

Electroporation-mediated DNA vaccination was performed with methods similar to those described by Jacob et al. [26]. C57BL/6 mice (5 per group) were injected in the tibialis muscle of the shaved hind leg. The appropriate concentration of DNA plasmid was diluted in a total volume of 20 μL of PBS. DNA injection was followed immediately by square wave electroporation at the injection site using a BTX830 (BTX Harvard Apparatus, Holliston, MA). A tweezers electrode was used to deliver eight pulses at 100 V for 20 ms. Vaccinated mice were boosted with the same dose and regimen on the contralateral leg 1 week later. Splenocytes were harvested 1 week after the last vaccination.

### Comparison Vaccinations

Peptide vaccination was performed using subcutaneous injection of 15 μg of OVA8 (aa257-264) in incomplete Freund's adjuvant (IFA, Difco Laboratories) (50% v/v), or 50 μg of OVA30 (aa241-270) in IFA in a total volume of 200 μL [27]. Protein-based vaccination was performed using subcutaneous injection of 50 μg of OVA protein in IFA similar to methods described previously [28]. Dendritic cell-based vaccination was performed using subcutaneous injection with 5 × 10^5 ^OVA8-pulsed bone marrow-derived dendritic cells (BMDC) similar to methods described previously [24]. Vaccinia-based vaccination was performed as described previously [29] using intraperitoneal injection with 1 × 10^7 ^pfu of OVA-expressing vaccinia viruses (OVA-VV) in 200 μL PBS. C57BL/6 mice (5 per group) were vaccinated with OVA8 in IFA, OVA30 in IFA, OVA protein in IFA, or OVA8-pulsed BMDCs. Vaccinated mice were boosted 1 week later at the same dose and regimen. At the same time of boost, a separate set of C57BL/6 mice were intraperitoneally injected with OVA-VV. Splenocytes were harvested 1 week after last vaccination.

### Intracellular cytokine staining and flow cytometry analysis

Pooled splenocytes from each vaccination group were incubated for 20 hours with 1 μg/ml of OVA SIINFEKL peptide or TERT198 peptide (aa198-205) (VGRNFTNL) [9] in the presence of GolgiPlug (BD Pharmingen, San Diego, CA). The stimulated splenocytes were washed once with FACScan buffer, stained with PE-conjugated monoclonal rat antimouse CD8a (clone 53.6.7), and subjected to intracellular cytokine staining using the Cytofix/Cytoperm kit according to the manufacturer's instructions (BD Pharmingen, San Diego, CA). Intracellular IFN-γ was stained with FITC-conjugated rat anti-mouse IFN-γ (clone XMG1.2). Flow cytometry analysis was performed using FACSCalibur with CELLQuest software (BD Biosciences, Mountain View, CA).

### In vivo tumor experiments

The OVA-expressing B16/OVA murine tumor model has been described previously [30]. 5-8 weeks old C57BL/6 mice (five mice per group) were injected with 1 × 10^5 ^B16/OVA tumor cells subcutaneously. 3 days after tumor cell inoculation, the tumor-bearing mice were treated with either 5 μg (total L1 protein amount) of HPV16-OVA, or HPV16-GFP pseudovirions via footpad injection. Tumor-bearing mice were boosted with the same regimen on day 10 and 17 after tumor challenge. Tumor-bearing mice without treatment were included as a control. Tumor growth was monitored twice a week. Tumor volumes were evaluated using the formula V(mm^3^) = 3.14[largest diameter × (perpendicular diameter)^2^]/6.

### Statistical analysis

Data expressed as means ± standard deviations (SD) are representative of at least two different experiments. Comparisons between individual data points were made by 2-tailed Student's *t *test. A *p *value < 0.05 was considered significant.

## List of Abbreviations

(BMDCs): Bone marrow-derived dendritic cells; (HPV): Human papillomavirus; (OVA): Ovalbumin; (psV): pseudovirions; (VLPs): virus-like particles.

## Competing interests

The authors declare that they have no competing interests.

## Authors' contributions

SP was involved in the design of the study and acquired and analyzed the data. SHC analyzed and interpreted the data. BM and TCW analyzed and interpreted the data and drafted the manuscript. CFH and TCW conceived and designed the study as well as provided general supervision of the research group. All authors gave final approval of the manuscript.
